# Editorial Notes

**Published:** 1931

**Authors:** 


### Editorial Notes

**Sir Vernon Wills.** THE dismay with which all Bristol heard that Sir Vernon Wills had died suddenly was nowhere felt more keenly than in hospital and university circles. Succeeding his father as President of the General Hospital, he had at once shown an interest in the work which did not limit itself to attendance at meetings, nor even to generous financial support. Both of these, indeed, he gave. In addition, however, he had formed his own views of the constructive side of hospital management, and approved of the new proposals for union. That he would have taken a leading part in the development of the Bristol School of Medicine can scarcely be doubted. At all events, we may record our appreciation of what he had already been able to accomplish, and recognize gratefully that "being made perfect in a little while he fulfilled long years."

**The General Hospital.** THE General Hospital has begun work in its new Out-patient Department and wards without any elaborate ceremony because of the death of its President. On 13th March, in the presence of the Committee and the Honorary Staff, Miss Hilda Wills formally declared the new premises open. Mrs. Wynne Wilson was also present. The replacement of the old Out-patient Department was long overdue, and the

Hospital was fortunately enabled by the munificence of the late Sir George Wills, Bart., to build a new one without incurring debt or having to make any public appeal. Built below "deck," the new department faces Commercial Road and Lower Guinea Street, and thus offers easy access to patients without bringing them through the rest of its premises. The new buildings comprise a Casualty Unit with Operating Theatre, an Out-patient Hall with rooms for the various departments, and two wards. One of these, a Children's Ward with sixteen cots, succeeds to that which was recently converted into a new operating theatre in the main building, which was formally opened by Lord Moynihan on 1st May, 1930. The Louisa Lucas Ward, given by Mr. Bernard Lucas, has at his request been devoted to the treatment of accident cases. It contains fourteen beds. These additions will set free in the old Out-patient Department space which has long been needed for the better housing of physiotherapy of various kinds and for an expansion of the X-ray Department.

It is hoped that the annexe which is to be built, in conjunction with the Royal Infirmary, at Winford will provide all that is needed in the shape of additional ward accommodation.

**National Council for Mental Hygiene.**

THE National Council for Mental Hygiene is holding its second biennial Conference from Wednesday to Friday, 27th May to 29th May, 1931, at the Central Hall, Westminster.

The subjects to be discussed are: The human factor in international problems; the human factor in relation to crime, in relation to industry, in relation to the social services, and in relation to education.

Tickets are issued at 7s. 6d. each, admitting holders to all sessions, or at 2s. each for the single sessions. Donations towards the organizing expenses will also be welcomed.

Tickets and all further particulars may be obtained from the Secretary, the National Council for Mental Hygiene, 78 Chandos House, Palmer Street, London, S.W.1.

